# A Retrospective Analysis of the Clinical Features of Inpatients With Epilepsy in the Ganzi Tibetan Autonomous Prefecture

**DOI:** 10.3389/fneur.2018.00891

**Published:** 2018-10-30

**Authors:** Jiani Chen, Xintong Wu, Yongqiao He, Sisi Li, Yongyi Deng, Jie Chen, Wenyu Fang, Zhamu Zeren, Jianmei Peng, Yingjuan Li, Jie Mu, Dong Zhou

**Affiliations:** ^1^Department of Neurology, West China Hospital, Sichuan University, Chengdu, China; ^2^Department of Neurology, Ganzi Tibetan Autonomous Prefecture People's Hospital, Kangding, China

**Keywords:** cerebral infection, clinical features and atiology, epilepsy, Ganzi Tibetan Autonomous Prefecture, retrospective study

## Abstract

**Background:** There is limited detailed clinical information for patients with epilepsy in Tibet. This study sought to provide data about the clinical features of epilepsy in the Ganzi Tibetan Autonomous Prefecture to improve strategies for epilepsy prevention and management in this region.

**Methods:** We reviewed the clinical record of patients with epilepsy in the Neurology Department, Ganzi Tibetan Autonomous Prefecture People's Hospital and compared the clinical features and compared it with control, from West China Hospital in Chengdu.

**Results:** This retrospective study included 165 patients with epilepsy admitted between January 2015 and February 2018. Majority of patients (97%) in this study had active epilepsy; 28.5% had generalized onset seizures and 68.5% had focal onset seizures. Fifty-four patients had received anti-epileptic drug (AED) treatment prior to hospitalization, however, 38 (70.4%) patients took the medication irregularly. The leading etiology of this cohort was head trauma (20.6%), followed by stroke (10.9%), neurocysticercosis (7.9%), brain hydatidosis (6.7%) and tuberculous infection (5.5%). Compared with in-patients in Chengdu, epilepsy in Ganzi was more frequently caused by infection (OR = 4.216, 95% CI, 2.124–8.367), including neurocysticercosis (OR = 29.301, 95% CI, 1.727–497.167) and brain hydatidosis (OR = 24.637, 95% CI, 1.439–421.670).

**Conclusions:** These data suggest that the control of cerebral infections, especially parasite infection, is essential for the prevention of epilepsy in the Ganzi Tibetan Autonomous Prefecture. Education of local primary doctors and patients about the literacy of epilepsy will enable better management of epilepsy in this population.

## Introduction

Epilepsy is one of the most common neurological disorders in the world that affects people of all nations and socioeconomic classes. The Tibetan area, which is on the Qinghai-Tibetan plateau, is ~4,000 m above sea level. There are very few studies of epilepsy in this area (1, 2). A survey done in one of the counties in Tibet found that the lifetime prevalence of convulsive epilepsy was ~2.5 per 1,000 with a treatment gap of up to 97%. No EEG or imaging data were available in this study (1). Another study summarized 180 cases of patients with epilepsy (PWE) and reported that the etiology was unknown in 145 cases (80.6%) and no EEG or imaging data were available (2). Detailed clinical information, such as MRI and EEG results in the Tibet area, remains limited.

This study sought to provide data about the clinical features of epilepsy in the Ganzi Tibetan Autonomous Prefecture. Ganzi Tibetan Autonomous Prefecture is on the South-eastern edge of the Qinghai-Tibetan plateau, in the Western part of Sichuan Province. The average altitude is 3,500 meters above sea level. Tibetan is the primary ethnic group. With a population of one million, Ganzi Tibetan Autonomous Prefecture is the second largest inhabited region of Tibetan people in China. Many locals earn their living by farming or yak herding (3).

In this study, clinical records of patients admitted to Ganzi Tibetan Autonomous Prefecture People's Hospital and diagnosed with epilepsy were retrospectively reviewed. The study aimed at providing an updated information on the clinical features of epilepsy in the Tibetan area to help improve strategies for epilepsy prevention and management in this region.

## Materials and methods

### Study location and population

The Ganzi Tibetan Autonomous Prefecture People's Hospital is in Kangding City, Sichuan province, the capital of the Ganzi Tibetan Autonomous Prefecture. It is the only hospital equipped with MRI and Video EEG in Ganzi. West China Hospital, a large tertiary referral center in West China, has been sending a neurologist to the Ganzi Tibetan Autonomous Prefecture People's Hospital every year since 2014 to live and work with the local neurologists.

### Study design

Patients with epilepsy that were admitted to the Neurology Department of Ganzi Tibetan Autonomous Prefecture People's Hospital, between January 2015 and February 2018, were retrospectively and consecutively included in this study. To explore the clinical features of inpatients with epilepsy in Ganzi, demographic and clinical data collected were compared with the data from West China Hospital in Chengdu (control group). Controls' data were retrieved from in-patient epilepsy database of West China Hospital in Chengdu City between June 2017 and February 2018. PWE in Ganzi were identified and matched for gender, age (±2 years) in the controls database. In cases where there were several matched controls, the one with early admission date would be included.

The following definitions were considered: epilepsy is defined according to the 2014 International League Against Epilepsy (ILAE) as two spontaneous seizures with one occurring within the last 6 months, or one seizure with an epileptiform discharge shown by EEG or an enduring relevant lesion shown by imaging (4, 5).

Status epilepticus (SE) is defined according to the 2015 ILAE as a condition resulting from the failure of the mechanisms responsible for seizure termination or initiation of mechanisms which lead to abnormally prolonged seizures. Convulsive SE is defined as more than 5 min of continuous seizure or two or more discrete seizures with incomplete recovery between them (6).

Patients were excluded if they reported symptomatic seizures occurring <2 weeks after the onset of acute cerebral insults and if they lived outside of the Ganzi Tibetan Autonomous Prefecture.

Controls were excluded if they reported symptomatic seizures occurring <2 weeks after the onset of acute cerebral insults.

### Socio-demographic status and clinical information

Data were collated retrospectively using a structured data collection form by reviewing the medical charts. All patients recruited had been diagnosed with epilepsy by two neurologists, from both the Ganzi Tibetan Autonomous Prefecture People's Hospital and West China Hospital. Differences in opinion were resolved by consensus. Demographic information included gender, age, marriage status, living area, education, job, and ethnicity were collected.

Clinical information was collected from all patients and the control. This included: age at the seizure onset, seizure frequency, and AEDs. The results of EEG and MRI/CT scans were reviewed and recorded. The MRI prescribed was a 1.5-T imaging system (Philips Achieva, Holland & Sonata, Siemens, Germany). Most patients in our clinic underwent neuroimaging as part of routine checks. Local or generalized slow waves in the EEG recording were considered non-specific abnormalities, while local or generalized paroxysms of sharp waves, spike waves, sharp and slow waves, or spike and slow waves were considered epileptiform discharges. Epileptic seizures were classified according to ILAE 2017 classification as focal seizures (focal aware seizures, focal impaired awareness seizures, or focal to bilateral tonic-clonic seizures), generalized seizures (non-motor, other motor or tonic-clonic), and unknown (7). Etiology was defined as the presence of remote brain injury that causes seizure episodes (8). Diagnosis was typically established on the basis of clinical history, past medical records, and laboratory results, and was further confirmed by neuro imaging. Etiology was classified according to ILAE 2017 classification as structural, infectious, genetic, metabolic, immune, and unknown etiology (7, 9). Specific etiologies were also listed, including neurocysticercosis, brain hydatidosis, stroke, head trauma, cerebral tumor, hippocampal sclerosis, cortical dysplasia, unclear intracranial space-occupying lesions, and so on.

The study was approved by the West China Hospital clinical trial and biomedical ethics committee and the ethics committee of the Ganzi Tibetan Autonomous Prefecture People's Hospital.

### Data analysis

Descriptive statistics of the patients and controls were conducted. Quantitative data were expressed as the mean ± SD. Qualitative data were summarized as proportions. A comparison of demographic and clinical data between the two groups was done using Student's *T*-tests/Mann Whitney *U* test for continuous variables and Chi-squared tests for categorical variables, respectively.

IBM SPSS 23.0 was used to perform all analyses. All of the tests were two-tailed, and *p*-values <0.05 were considered to be statistically significant.

## Results

### Demographic data of the participants

One hundred and sixty-seven PWE were admitted to the Neurology Department of the Ganzi Tibetan Autonomous Prefecture People's Hospital from January 2015 to February 2018. Out of these, two patients were excluded because they were travelers living outside the Ganzi Tibetan Autonomous Prefecture. Hence, 165 patients were included in the study.

Our patient population included 96 (58.2%) men and 69 (41.8%) women. Other demographic features are listed in Table 1. The mean patient age was 39.4 ± 16.8 years. Forty-one (24.8%) patients lived in an urban location, and 124 (75.2%) lived in a rural/pastoral location. Most patients were Tibetan (*n* = 128, 77.6%) or Han Chinese (*n* = 34, 20.6%). The number of illiterate patients was 104 (63.0%), and 60.6% of the total patients were farmers.

**Table 1:** Demographic features of PWEs in the neurology department of Ganzi people's hospital and control group from West China hospital.

**Variable**	**PWEs in Ganzi *N* = 165**	**Control group *N* = 165**	***P*-value**
Gender, *n* (%)	–	–	1.000
Female	69 (41.8%)	69(41.8%)	–
Male	96 (58.2%)	96 (58.2%)	–
Age (year, mean ± SD)	39.4 ± 16.8	40.0 ± 17.0	0.721
Age group, *n* (%)	–	–	0.996
≤14 years	5 (3.0%)	5 (3.0%)	–
15–44 years	99 (60.0%)	97 (58.8%)	–
45–59 years	37 (22.5%)	38 (23.0%)	–
≥60 years	24 (14.5%)	25 (15.2%)	–
Marriage, *n* (%)	–	–	0.729
Married	109 (66.1%)	106 (64.2%)	–
Single	56 (33.9%)	59 (35.8%)	–
Living area, *n* (%)	–	–	0.000^*^
Urban	41 (24.8%)	105 (63.6%)	–
Rural/pastoral	124 (75.2%)	60 (36.4%)	–
Education status, *n* (%)	–	–	0.000^*^
Illiterate	104 (63.0%)	2 (1.2%)	–
Primary school and middle school	38 (23.0%)	77 (46.7%)	–
High school	15 (9.1%)	37 (22.4%)	–
College	8 (4.8%)	49 (29.7%)	–
Job, *n* (%)	–	–	0.000^*^
Not employed^b^	9 (5.5%)	41 (24.8%)	–
Student	17 (10.3%)	26 (15.8%)	–
Farmer	100 (60.6%)	30 (18.2%)	–
Employment	21 (12.7%)	46 (27.8%)	–
Retirement	7 (4.2%)	11 (6.7%)	–
Other	11 (6.7%)	11 (6.7%)	–
Ethnic Group, *n* (%)	–	–	0.000^*^^a^
Han	34 (20.6%)	158 (95.8%)	–
Tibetan	128 (77.6%)	5 (3.0%)	–
Yi	3 (1.8%)	2 (1.2%)	–

* *P < 0.05*.

a *Fisher exact test*.

b *Including housewife*.

A comparison of the demographic characteristics of the in-patients in Ganzi and gender and age-matched controls in Chengdu was done (Table 1). The comparison indicates that there were more patients in Ganzi who lived in rural/pasture areas compared to the controls (75.2 vs. 36.4%, *p* < 0.001) as well as lower education levels (the illiterate up to 63.0 vs. 1.2%, *p* < 0.001). There was also a significant difference in job groups (*p* < 0.001) and ethnic group distribution (77.6% Tibetan in Ganzi patients, *p* < 0.001). No differences were detected for the age, gender, and marital status.

### Clinical features

The mean age at the time of seizure onset was 34.5 ± 18.0 years. No significant difference was noted when this was compared with the data from the control group (Table 2). In addition, there were more patients newly diagnosed with epilepsy 104 (63.0%) compared to the control group (63.0 vs. 33.3%, *p* < 0.001). The different seizure types were as follows: focal aware seizures (*n* = 5, 3.0%); focal impaired awareness seizures (*n* = 12, 7.3%); focal to bilateral tonic-clonic seizures (*n* = 96, 58.2%); and generalized tonic-clonic seizures (*n* = 46, 27.9%). Thirty-three (20%) out of 165 patients were diagnosed with SE, while 2 (1.2%) died following prolonged SE. No significant difference in seizure type fund between groups (focal/generalized/unknown, *p* = 0.087). Most (98.8%) patients underwent an MRI or CT scan; abnormalities were noted in 102 (61.8%) patients. These were more than those in the control group (61.8 vs. 38.2%, *p* < 0.001). EEG or video EEG was performed in 51 (30.9%) patients. These were less than those in the control group (30.9 vs. 91.5%, *p* < 0.001). The results of the EEG studies revealed that 27 (52.9%) and 19 (37.3%) patients showed epileptiform discharge and normal EEG, respectively.

**Table 2:** Clinical features of PWEs in neurology department of Ganzi people's hospital and control group from West China hospital.

**Variable**	**PWEs in Ganzi *N* = 165**	**Control group *N* = 165**	***P*-value**
Age of onset (year, mean ± SD)	34.5 ± 18.0	31.9 ± 19.0	0.208
Admitted for status epilepticus, *n* (%)	33 (20.0%)	17 (10.3%)	0.014^*^
Death after status epilepticus	2 (6.1%)	0 (0%)	0.517^a^
Newly diagnosis	104 (63.0%)	55 (33.3%)	0.000^*^
Seizure type, *n* (%)	–	–	0.087^*^^b^
Generalized onset	47 (28.5%)	66 (40%)	–
Tonic-clonic	46 (27.9%)	65 (39.4%)	–
Nonmotor	1 (0.6%)	1 (0.6%)	–
Focal onset	113 (68.5%)	95 (57.6%)	–
Impaired awareness	12 (7.3%)	32 (19.4%)	–
Aware	5 (3.0%)	7 (4.2%)	–
Focal to bilateral tonic-clonic	96 (58.2%)	56 (33.9%)	–
Unknown onset	5 (3.0%)	4 (2.4%)	–
EEG, *n* (%)	51 (30.9%)	151 (91.5%)	0.001^*^
Normal	19 (37.3%)	87 (57.6%)	–
Non-specific abnormality	5 (9.8%)	26 (17.2%)	–
Epileptiform discharge	27 (52.9%)	38 (25.2%)	–
MRI/CT scan, *n* (%)	–	–	0.000^*^
Abnormal	102 (61.8%)	63 (38.2%)	–
Normal	61 (37.0%)	100 (60.6%)	–
Anti-epileptic drugs at the time of discharge, *n* (%)	–	–	0.000^*^
No treatment	18 (10.9%)	24 (14.5%)	–
Mono-therapy	129 (78.2%)	90 (54.5%)	–
Poly-therapy	18 (10.9%)	51 (31.0%)	–

* *P < 0.05*.

a *Haldane–Anscombe correction*.

b *Fisher exact test*.

The data collected also revealed that 61 (36.9%) patients had been diagnosed with epilepsy prior to hospitalization with 54 of these patients receiving prescriptions for AEDs. However, 38 (70.4%) patients reported irregular use of medication, significantly higher than the control group (70.4 vs. 22.5%, *p* < 0.001). Moreover, 5 cases of SE were precipitated by nonadherence to the medication regime. The mean duration of status epilepticus before admission was 15.5 h (range 0.5–48). Most patients in this study (97.0%) had active epilepsy (seizure free <1 year) (10).

### Etiology

The etiological factors of epilepsy in Ganzi are summarized in Table 3. With the ILAE 2017 classification, 39.4% had a structural etiology, 24.8% had an infectious etiology, 4.8% had a presumed genetic cause, and 29.7% was classified as unknown.

**Table 3:** General etiologic features of the epilepsy patients.

**Etiology classification**	**PWEs in Ganzi *N* = 165**	**Control group *N* = 165**	**OR (95% CI)**
Infectious	41 (24.8%)	12 (7.3%)	4.216 (2.124–8.367)^*^
Neurocysticercosis	13 (7.9%)	0 (0.0%)	29.301 (1.727–497.167)^*^^a^
Brain hydatidosis	11 (6.7%)	0 (0.0%)	24.637 (1.439–421.670)^*^^a^
Tuberculous	9 (5.5%)	3 (1.8%)	3.115 (0.828–11.721)
Viral infection	6 (3.6%)	8 (4.8%)	0.740 (0.251–2.183)
Brain abcess	2 (1.2%)	1 (0.6%)	1.974 (0.177–22.001)
Structural	65 (39.4%)	60 (36.4%)	1.137 (0.728–1.775)
Head trauma	34 (20.6%)	19 (11.5%)	1.994 (1.084–3.666)^*^
Stroke	18 (10.9%)	11 (6.7%)	1.714 (0.783–3.752)
Ischemic	11 (6.7%)	5 (3.0%)	2.285 (0.776–6.731)
Hemorrhage	7 (4.2%)	6 (3.6%)	1.174 (0.386–3.571)
Cerebral tumor	3 (1.8%)	6 (3.6%)	0.490 (0.120–1.996)
Hippocampus sclerosis	1 (0.6%)	10 (6.0%)	0.094 (0.012–0.747)^*^
Cortical dysplasia	4 (2.4%)	8 (4.8%)	0.487 (0.143–1.651)
Unclear intracranial space-occupying lesions	5 (3.0%)	3 (1.8%)	1.687 (0.396–7.179)
Other structural changes	0 (0.0%)	3 (1.8%)	0.143 (0.007–2.737)^a^
Metabolic	0 (0.0%)	0 (0.0%)	1.000 (0.019–50.699)^a^
Immune^b^	2 (1.2%)	3 (1.8%)	0.662 (0.109–4.018)
Presumed genetic cause^c^	8 (4.8%)	17 (10.3%)	0.443 (0.186–1.058)
Unknown	49 (29.7%)	73 (44.2%)	0.532 (0.338–0.838)^*^

* *P < 0.05*.

a *Haldane–Anscombe correction*.

b *Autoimmune encephalitis*.

c *Included Genetic Generalized Epilepsies*.

As to specific causes of epilepsy, the leading cause was head trauma (*n* = 34, 20.6%), followed by stroke (*n* = 18, 10.9%), neurocysticercosis (*n* = 13, 7.9%), brain hydatidosis (*n* = 11, 6.7%) and tuberculous infection (*n* = 9, 5.5%). Other etiologies included presumed genetic cause (*n* = 8, 4.8%), viral infection (*n* = 6, 3.6%) and unclear intracranial space-occupying lesions (*n* = 5, 3.0%). Less common etiologies included tumor (*n* = 3, 1.8%), cortical dysplasia (*n* = 4, 2.4%), and hippocampal sclerosis (*n* = 1, 0.6%).

When compared with the control group, PWEs in Ganzi significantly had more frequent infectious etiology (OR = 4.216, 95% CI, 2.124–8.367). And epilepsy in Ganzi was caused more frequently by head trauma (OR = 1.994, 95% CI, 1.084–3.666), neurocysticercosis (OR = 29.301, 95% CI, 1.727–497.167) and brain hydatidosis (OR = 24.637, 95% CI, 1.439–421.670). Epilepsy in Ganzi was less frequently caused by hippocampus sclerosis (OR = 0.094, 95% CI, 0.012–0.747). The percentage of patients with unknown etiology in Ganzi was lower compared with the control group (OR = 0.532, 95% CI, 0.338–0.838).

## Discussion

The present study focused on the clinical features of epilepsy in the Ganzi Tibetan Autonomous Prefecture, which is currently under-studied. The aim is to obtain clinical information that would lead to improved strategies in prevention, management, education, and treatment of epilepsy in this region.

### Status epilepticus

Thirty-three PWE who were admitted to the hospital had SE. Two of them (6.1%) died in the hospital. The reported overall mortality is ~20% in the first month after SE (11). However, in SE without provoked factors, the reported rate of death is ~10% (11, 12). In the current study, we excluded acute symptomatic cases from our cohort. For the two fatalities recorded, it was noted that the SE lasted for more than 1 day before proper treatment was initiated. In addition, some people living in the remote county of the Ganzi Autonomous Tibetan Prefecture would require 2 days drive to reach the People's Hospital. This may have been the reason for delayed diagnosis and treatment. The mean duration of SE before admission was 15.5 h (range 0.5–48). It is therefore important that primary hospital physicians have to provide early diagnosis and control of SE. A survey done on attitudes and management practices of epilepsy among 100 primary practitioners in Ganzi, 2017, revealed that 49% did not know how to treat SE (unpublished data). It is therefore important to train primary practitioners and equip them with skills to control these cases.

### AED, seizure control and medication compliance

The findings of this study indicate that 63.0% of patients were newly diagnosed. Most of the patients who had been diagnosed with epilepsy previously had received AED treatment prior to hospital admission (54 out of 61 patients). However, 38 (70.4%) out of 54 patients reported irregular treatment. Majority of the of the 165 patients (97.0%), reported having active epilepsy (seizure free <1 year) (10). Previous studies have shown that medication compliance is poor in this region. One study reported that 87.5% of patients were not taking regular treatment (1). The factors that may contribute to non-compliance include: patients' perception of the importance of the medication or lack of regular doctor's appointments (13). Furthermore, many patients turn to traditional Tibetan treatment in the Tibet Autonomous Region (2). A survey on the attitudes toward epilepsy among the general population in Ganzi, 2017, revealed that up to 67% of patients preferred traditional Tibetan medicine because they thought it would work (unpublished data). The current study revealed that 78.2% of patients had mono-therapy prescriptions when discharged from Ganzi People's Hospital. However, follow up was not done to ascertain their compliance to the medication. Medication reminders (14) and educational programmes (13) may increase compliance in these patients.

### Etiological factors and prevention of parasite infection

Two previous population-based studies from the Tibet Autonomous Region did not conclusively describe the etiology of epilepsy in the patient populations studied. This may be due to the unavailability of EEG and CT/MRI data (1, 2). One study reported that seven and three patients developed epilepsy due to head trauma and neurocysticercosis, respectively (1). Another study (2) identified one patient with head trauma, one with neurocysticercosis, and 35 patients with seizures associated with heavy drinking or alcohol withdrawal. However, about 80% of patients in both studies have unknown etiologies. A strength of the current study is that most patients in our study underwent CT/MRI scans, hence, we were able to identify more etiological factors than previous study. The leading cause in this study was head trauma (20.6%), followed by stroke (10.9%), neurocysticercosis (7.9%), brain hydatidosis (6.7%), and tuberculous infection (5.5%). Head trauma, stroke, cerebral infection are common causes for epilepsy. In the epidemiological survey of epilepsy in Rochester, Minnesota described the following etiological factors, such as cerebrovascular diseases (6%), neurologic deficits from birth (5%), trauma (5%), and infection (4%) (15). Head trauma, intracranial infection, and cerebrovascular diseases were the leading causes of epilepsy in a large population-based study in China in the 1980s (16). In comparison, the inpatient sample in Ganzi Tibetan area had parasite infection of the brain accounting for about 14.6% percent of all epilepsy patinets in this sample. This was higher than the age and gender matched control group. It was also significantly higher when compared with another etiology study in Chengdu (neurocysticercosis: 7.9 vs. 1.3%, OR 6.271, 95% CI 2.808–14.004; Brain hydatidosis: 6.7 vs. 0%, OR 132.864, 95% CI 7.788–2,266.400) (17). The adult of tapeworm live in the human intestine, and the intermediate hosts are pigs or yaks often found in this area. Humans often develop neurocysticercosis when they ingest raw meat containing cysts (18, 19). The adult of Echinococcus live in dogs or Tibetan foxes. Yaks serve as the intermediate hosts. Humans often get infected when they eat food contaminated with echinococcus eggs. Hydatidosis could occur in liver, lung or brain (20). Both cysticercosis and hydatidosis are endemic diseases in Ganzi. One study has shown that 22.5% of the study participants had positive serology positive for cysticercosis in Yajiang County, Ganzi. Half of the seropositive patients were noted to experience seizures (18). Approximately an eighth (12.9%) of screened people were infected with hydatidosis in Shiqu County, Ganzi (21).

In the high pastures, many locals still live a traditionally pastoralism and semi-nomadism lifestyles, keeping dogs for herding animals. The domestic dog appears to be a key risk factor for both cystic and alveolar echinococcosis (20). Rates of echinococcosis infection in domestic dogs have been reported to be 5–15% in Tibetan area (22). Controlling the number of dogs and monthly praziquantel dog dosing programme is essential in preventing echinococcosis (20). Improvement of hygiene and promotion of hand-washing could also help in reducing infections (18).

Human taeniasis is acquired by eating uncooked or poorly cooked pork or beef infected with the larval form of the human taeniid tapeworms (19), there is a habitual consumption of undercooked beef and air-dried raw beef in this Tibetan community. Strategies to educate the locals on proper cooking of meat are important in the prevention of taeniasis (18).

Suprisely, the percentage of patients with unknown etiology was higher in the control group, suggesting that in-patients in Ganzi might have more identifiable seizure etiologies, such as infectious and structural changes. Hydatidosis is a critical and often fatal disease if left untreated (23). This study therefore proposes that all patients in this area with a new diagnosis of epilepsy should undergo a hydatidosis screen test.

The average altitude is 3,500 meters above sea level in Ganzi Tibetan Autonomous Prefecture. Excepted the two study in Tibet Autonomous Region which had been described above (1, 2), epidemiology of epilepsy in high altitude region was few (24), though there have been multiple case reports of seizures occurring in non-epileptic individuals and well-controlled epileptic patient at altitude (25–27). Still, the effect of altitude on the seizure threshold has not been studied in depth. This issue need to be explored more in future studies.

### Gender and age

Our study cohort comprised 58.2% male patients (male: female, 1.39:1). A door-to-door survey of six cities in China in 1987 regarding PWE reported a male-to-female ratio of 1.15:1 (16). An additional population-based survey in the Tibet Autonomous Region reported a male-to-female ratio of 1.73:1 (2). Our study may have a higher number of man than the number that is in the general population; however, our ratio is similar to those observed in previous studies that note a greater number of male patients than female patients.

Mean age of patients was 39.4 ± 16.8 years. The mean onset age was 34.5 ± 18.0. Our age group distribution was similar to that observed in previous population-based surveys in Tibetan area, with over half of the patients in the 15–44 years age group (1, 28). However, the predominant middle-age group in this study is unusual as compared to the bimodal age distribution reported in the literature (15, 28). This may be because many of them had infectious etiology, as the mean onset age of infectious etiology was 34.3 ± 15.1. Besides, Children <14 years of age were directed to the pediatrics department and hence most were not included in this study.

### Semiology

In this cohort 28.5% of patients had generalized onset seizures and 68.5% of patients had focal onset seizures. Generalized tonic-clonic seizures and focal to bilateral tonic-clonic seizures were the most commonly observed seizure type. This result is consistent with the findings of previous studies in the Tibet Autonomous Region (1, 2). However, the EEG application rate for epilepsy diagnosis was 30.9% in our study, which is significantly lower than the control group in Chengdu. This may compromise the accuracy of diagnosis, in particular for seizure semiology. Though the EEG rate in the current study is higher than that in previous research conducted in the Tibetan area (1, 2), it would be beneficial to have a much higher EEG rate in future studies.

### Study limitations

This is a hospital-based cohort and a retrospective study. Children younger than 14 years old, who were typically admitted to the Pediatric Department, were not included. This admission bias and our study results may not apply to the entire population in the Ganzi region. In addition, we found that 30.9% patients underwent EEG for epilepsy diagnosis; therefore, the accuracy of seizure semiology may be reduced. Nonetheless, the present study provides useful imformation for future studies and can guide the development of strategies for managing epilepsy in the Tibetan region.

## Conclusions

This study suggests that the control of endemic infectious diseases in local regions, such as cysticercosis, and hydatidosis, is important for better prevention of epilepsy. In addition, education about seizures for local primary doctors and patients will enable better management of epilepsy in this population.

## Data availability statement

The raw data supporting the conclusions of this manuscript will be made available by the authors, without undue reservation, to any qualified researcher.

## Author contributions

DZ designed this study. JiaC and XW carried out this study; those two authors collected data, analyzed data, and contributed equally to this study. JiaC, YH, YD, JieC, WF, and YL helped in data collecting. SL helped analyzed results. ZZ, JP, and JM helped in EEG data analysis, JiaC wrote the draft and XW revised the final version.

### Conflict of interest statement

The authors declare that the research was conducted in the absence of any commercial or financial relationships that could be construed as a potential conflict of interest.
